# The Role of the FMN-Domain of Human Cytochrome P450 Oxidoreductase in Its Promiscuous Interactions With Structurally Diverse Redox Partners

**DOI:** 10.3389/fphar.2020.00299

**Published:** 2020-03-18

**Authors:** Francisco Esteves, Diana Campelo, Bruno Costa Gomes, Philippe Urban, Sophie Bozonnet, Thomas Lautier, José Rueff, Gilles Truan, Michel Kranendonk

**Affiliations:** ^1^Centre for Toxicogenomics and Human Health (ToxOmics), Genetics, Oncology and Huma Toxicology, NOVA Medical School, Faculty of Medical Sciences, Universidade NOVA de Lisboa, Lisbon, Portugal; ^2^Centre National de la Recherche, Scientifique, Institut National de la Recherche Agronomique, Institut National des Sciences Appliqu es de Toulouse, Toulouse Biotechnology Institute, Universit de Toulouse, Toulouse, France

**Keywords:** NADPH cytochrome P450 oxidoreductase (CPR), cytochrome P450 monooxygenases (CYP), FMN-domain, binding motifs, redox partner, protein–protein interaction, electron transfer

## Abstract

NADPH cytochrome P450 oxidoreductase (CPR) is the obligatory electron supplier that sustains the activity of microsomal cytochrome P450 (CYP) enzymes. The variant nature of the isoform-specific proximal interface of microsomal CYPs indicates that CPR is capable of multiple degenerated interactions with CYPs for electron transfer, through different binding mechanisms, and which are still not well-understood. Recently, we showed that CPR dynamics allows formation of open conformations that can be sampled by its structurally diverse redox partners in a CYP-isoform dependent manner. To further investigate the role of the CPR FMN-domain in effective binding of CPR to its diverse acceptors and to clarify the underlying molecular mechanisms, five different CPR-FMN-domain random mutant libraries were created. These libraries were screened for mutants with increased activity when combined with specific CYP-isoforms. Seven CPR-FMN-domain mutants were identified, supporting a gain in activity for CYP1A2 (P117H, G144C, A229T), 2A6 (P117L/L125V, G175D, H183Y), or 3A4 (N151D). Effects were evaluated using extended enzyme kinetic analysis, cytochrome *b*_5_ competition, ionic strength effect on CYP activity, and structural analysis. Mutated residues were located either in or adjacent to several acidic amino acid stretches – formerly indicated to be involved in CPR:CYP interactions – or close to two tyrosine residues suggested to be involved in FMN binding. Several of the identified positions co-localize with mutations found in naturally occurring CPR variants that were previously shown to cause CYP-isoform-dependent effects. The mutations do not seem to significantly alter the geometry of the FMN-domain but are likely to cause very subtle alterations leading to improved interaction with a specific CYP. Overall, these data suggest that CYPs interact with CPR using an isoform specific combination of several binding motifs of the FMN-domain.

## Introduction

NADPH cytochrome P450 oxidoreductase (CPR) is the crucial electron donor of all microsomal cytochrome P450s (CYPs), involved in drug and xenobiotic metabolism, but also in other key biochemical processes such as cholesterol and fatty acid homeostasis and steroid hormone biosynthesis (Guengerich, 2015). Moreover, CPR is also the unique electron donor of more structurally divergent enzymes such as heme oxygenase and squalene monooxygenase (Ellis et al., 2009; Pandey and Flück, 2013; Waskell and Kim, 2015). The interaction between CPR and microsomal CYPs promotes a two electron transfer (ET) from NADPH through the CPR cofactors FAD (reductase) and FMN (transporter) to the heme iron of CYP (reviewed in, Waskell and Kim, 2015). CPR contains a hydrophobic N-terminal transmembrane segment, responsible for its association and orientation in the endoplasmic reticulum (ER). The highly flexible hinge region which links the FMN-binding domain to the FAD/NADPH-binding domain, is involved in the transition between CPR’s closed/locked (inter-flavin electron transfer) and open/unlocked (electron donation) conformations (Aigrain et al., 2009; Ellis et al., 2009; Hamdane et al., 2009; Sündermann and Oostenbrink, 2013; Frances et al., 2015; Campelo et al., 2017). The hinge region has been suggested to be largely responsible for the formation of diverse multiple open conformations, involved in the formation of productive enzyme complexes, in an apparent CYP isoform dependent manner (Campelo et al., 2018).

It has been a longstanding and intriguing issue how CPR is able to interact and sustain the activity of so many structurally diverse partners in its ET function (Kandel and Lampe, 2014; Hlavica, 2015). Charge pairing and van der Waal’s interactions have been implicated in this affinity sampling, indicating that both electrostatic and hydrophobic interactions are to be considered necessary for complex formation (Waskell and Kim, 2015). The importance of affinity in the formation of an effective enzyme complex is emphasized by the *in vivo* existence of CPR:CYP stoichiometry’s in favor of CYP, implying competition between individual CYP isoforms in binding to CPR in the ER (Kranendonk et al., 2008; Campelo et al., 2018). Therefore, the effective ET from CPR to structurally diverse redox partners seems to be enabled by: (1) the existence of multiple open conformations, the result of CPR’s open/closed dynamics, that can be sampled by its diverse redox partners; (2) affinity probing of these open conformers, guided by CYP-isoform specific affinity for the FMN-domain of CPR, to achieve effective docking of acceptors with CPR. Additionally, several earlier studies have suggested that the CPR transmembrane segment, mostly helical, could be implied in the preliminary association of the CPR with CYPs, mediated by their respective N-terminal transmembrane segments (Black et al., 1979; Gum and Strobel, 1981; Black and Coon, 1982; Bernhardt et al., 1983; Miyamoto et al., 2015). Moreover, the role of lipids and membrane anchoring of CPR and CYP have been recently confirmed to be of importance in the effectiveness of CPR’s ET (Barnaba et al., 2017; Campelo et al., 2017; Denisov and Sligar, 2017; Barnaba and Ramamoorthy, 2018).

The FMN-domain acts as the interface for ET to all CPR’s structurally diverse redox partners, including the 50 human microsomal CYP isoforms (Gutierrez et al., 2002; Hlavica and Lehnerer, 2010; Kandel and Lampe, 2014). This domain is highly conserved across species (Pandey and Flück, 2013) and it consists of a five-stranded parallel β-sheet in the core fold, flanked by α-helices with the FMN cofactor positioned at the tip of the C-terminal side of the β-sheet (reviewed in, Hlavica, 2015). The FMN-domain has several conserved patches of acidic residues on its solvent exposed surface, suggested to be involved in the electrostatic interaction with its ET partners (reviewed in, Hlavica, 2015; Waskell and Kim, 2015). Several studies questioned the role of specific residues involved in FMN binding (Shen et al., 1989; Marohnic et al., 2010) and in the interaction of CPR with its redox partners (Strobel et al., 1989; Nadler and Strobel, 1991; Shen and Kasper, 1995; Bridges et al., 1998; Hamdane et al., 2009). In these works CPR mutants, produced by site directed mutagenesis or chemically modified CPR variants, were used in structural and functional analysis of residues of the CPR-FMN domain. The importance of specific residues in the vicinity of the FMN binding domain was demonstrated as well as the role of acidic amino acids in CPR’s binding to its electron acceptors, however identification of these residues was dependent on which CPR: acceptor couple was studied (Hlavica et al., 2003). Three distinct clusters of acidic residues were indicated to be involved in CPR’s interaction with multiple redox-partners (Shen and Strobel, 1994; Zhao et al., 1999). Electrostatic potential surface analysis revealed basic residues on the proximal side of CYPs (Waskell and Kim, 2015). These are thought to steer the geometrically convex FMN-domain of CPR through its negatively charged patches to CYP’s concave docking site near the heme moiety (Williams et al., 2000; Hlavica and Lehnerer, 2010). However, microsomal CYPs do not demonstrate signature sequences of these basic residues at their proximal side, a feature displayed by mitochondrial CYPs in the interaction with their redox partner iron–sulfur protein adrenodoxin (Pikuleva et al., 1999). This suggests that CPR interacts with microsomal CYPs in an isoform-dependent manner, which we demonstrated recently (Campelo et al., 2018). Thus CPR has a degenerated binding site for interaction with its diverse electron acceptors, but besides the suggested involvement of the conserved negatively charged patches, it is so far unclear what features of the FMN-domain enable CPR to interact with so many structural diverse redox partners in its ET function.

The present study describes the search for structural features of CPR’s FMN-domain, responsible for its degenerated interaction with CYPs, for effective sampling of open conformations. This was accomplished through the isolation and study of FMN-domain mutants selected for increased CYP activity when combined with specific isoforms. These mutations, expected to lead to improved interaction of CPR with these CYPs, should be informative on the localization and nature of features required for this “one-serve-all“-type of interaction. Random mutation libraries of the FMN-domain in full length human CPR were created, with increasing mutation frequencies. Each of these libraries was combined with three representative human drug metabolizing CYP isoforms (Guengerich, 2015), phylogenetically apart, namely CYP1A2, 2A6, or 3A4^1^. Mutant CPR-pools were co-expressed with each of these three CYPs in a dedicated bacterial cell model and screened for increased CYP-activity, using our recently developed whole-cell high-throughput activity assay (Esteves et al., 2018). Using membrane fractions of selected clones, detailed enzyme kinetic analysis, study of ionic strength effect and cytochrome *b*_5_ (CYB5) competition were performed, to confirm improved interaction. Structural analyses were carried out to rationalize the role of the mutated FMN-domain residues in the CPR:CYP degenerate interaction. Collectively, our data seems to indicate that each CYP interacts with CPR in a isoform specific manner, involving specific combinations of binding motifs of the FMN-domain which were formerly identified to be involved in the CPR:CYP interactions.

## Materials and Methods

### Reagents

L-Arginine, thiamine, chloramphenicol, ampicillin, kanamycin sulfate, isopropyl β-D-thiogalactoside (IPTG) (dioxane-free), δ-aminolevulinic acid, glucose 6-phosphate, glucose 6-phosphate dehydrogenase, nicotinamide adenine dinucleotide phosphate (NADP^+^ and NADPH) were obtained from Sigma-Aldrich (St. Louis, MO, United States). LB Broth, bacto tryptone and bacto peptone were purchased from BD Biosciences (San Jose, CA, United States). Bacto yeast extract was obtained from Formedium (Norwich, United Kingdom). Ethoxyresorufin, methoxyresorufine and coumarin were obtained from BD Biosciences (San Jose, CA, United States) and dibenzylfluorescein from Santa Cruz Biotechnology (Santa Cruz, CA, United States). Resorufin, 7-hydroxy coumarin, fluorescein and dichlorophenolindophenol (DCPIP) were obtained from Sigma-Aldrich (St. Louis, MO, United States). A polyclonal antibody from rabbit serum raised against recombinant human CPR obtained from Genetex (Irvine, CA, United States) was used for immune-detection of the membrane-bound CPR. All other chemicals and solvents were of the highest grade commercially available.

### Construction of the CPR-FMN-Domain Mutant-Libraries

CPR mutant pools were constructed with controlled random mutation rates targeting the FMN-domain of CPR. Random mutants were produced using as a template the sub-cloning vector pUC_POR, containing the initial segment (1–1269 bp) of human *POR* cDNA (based on NCBI sequence NM_000941.3, encoding the CPR consensus protein sequence NP_000932.3). Error-prone PCR (epPCR) (McCullum et al., 2010) was used for random mutagenesis of the FMN-domain sequence using GeneMorph II Random Mutagenesis Kit (Agilent, Santa Clara, CA, United States), with primers AdRandom POR Fw and AdRandom POR Rv (see Supplementary Table S1), which yielded a 822 bp fragment encompassing the FMN-domain. Error-prone PCR reactions were as follows: 30 cycles at 95°C for 30 s, 55°C for 30 s, and 72°C for 1 min, and a final extension at 72°C for 10 min (GeneAmp PCR System 9700; Applied Biosystems, Foster City, CA, United States), using 0.5 μM of each primer, pUC_POR as template (ranging from 0.1 to 1.9 μg), 2.5 U Mutazyme II DNA polymerase (Agilent), 1x reaction buffer (Mutazyme II reaction buffer; Agilent), 0.2 mM of each deoxynucleoside triphosphates (dNTPs) (Thermo Fisher Scientific, Waltham, MA, United States), in a 50 μl final reaction volume. CPR-FMN mutant pools were characterized for their mutation frequencies (mutant/FMN-domain fragment) by direct sequencing of the epPCR amplicons of the *POR* fragments, with primers AdRandom POR Fw and AdRandom POR Rv. Sequences were aligned and analyzed (position-numbering was based on NCBI consensus sequences NM_000941.3) using BLAST (version 2.3) (NCBI) and MultAlin (French National Institute for Agricultural Research, Centre Toulouse Midi-Pyrénées). Five different libraries were generated with increasing mutation frequencies (see Supplementary Table S2).

The five pools of the mutated FMN-domain segments present in pUC_POR were cloned into the human full-length wild type CPR expression vector pLCM_POR_*wt*_ (Kranendonk et al., 2008), through *Eco*RI + *Aat*II digestion followed by ligation with T4 DNA Ligase (Rapid DNA Ligation Kit, Thermo Fisher Scientific). Alternatively, mutated fragments were used as mega-primers and cloned back into plasmid pLCM_POR using mega-primer PCR of the whole plasmid (mwPCR) (Miyazaki, 2011), followed by purification (GeneJET PCR Purification Kit, Thermo Fisher Scientific) and *Dpn*I treatment (Thermo Fisher Scientific). The five FMN-domain mutant-libraries, harbored in the pLCM_POR plasmid were used to transform *E. coli* PD301 (Duarte et al., 2005, 2007), already containing the expression vector (pCWori) (Fisher et al., 1992) for human CYP1A2, 2A6, or 3A4 (expressed in N-terminal modified forms), through standard electroporation procedures, creating a total of 15 different libraries (Observation: from here on clones containing CPR with mutations in the FMN-domain, combined with a human CYP present in BTC bacteria are simply designated as CPR_*mut*_/CYP versus when containing wild type CPR: CPR_*wt*_/CYP).

CPR_*mut*_/CYP clones were picked from overnight grown LB agar-plates supplemented with chloramphenicol 10 μg/L, ampicillin 50 μg/L and kanamycin 15 μg/L, and transferred to single wells of flat-bottom 96-well microplates (CoStar, Washington, DC, United States), previously filled with 200 μl LB broth supplemented with antibiotics (see above). This procedure was partially executed with the use of a Biomek 2000 Laboratory Automation Workstation (Beckman Coulter; Brea, CA, United States) and the Kbiosystems K2 automated colony picker system (Kbiosystems; Basildon, Essex, United Kingdom). Culture microplates were incubated at 37°C, for 16 h with agitation (250 rpm), and subsequently supplemented with 10% glycerol and stored at −80°C.

### High-Throughput Screening of CPR-FMN-Domain Mutant-Libraries

Stored (−80°C) cultures were transferred by replicate plating to new flat-bottom 96-well microplates, previously filled with 200 μl LB broth supplemented with antibiotics (see above) and incubated at 37°C, for 16 h, at 250 rpm. Subsequently, 50 μl of the grown cultures was used to inoculate a 96-deep well plate (VWR, Radnor, PA, United States), previously filled with 1400 μl TB medium, pH 7.5 (except for the CYP3A4 containing strains cultured at pH 6.8), supplemented with designated antibiotics (see above), 2 g/L peptone, 1 mg/L thiamine, 0.04% (v/v) trace elements solution, IPTG 0.2 mM, δ-aminolevulinic acid 0.05 mM (CYP1A2 expressing clones) or 0.1 mM (CYP2A6 and CYP3A4 clones). Cultures were grown at 27°C, at 310 rpm agitation for 22 h, using gas-permeable membranes (Greiner bio-one, Kremsmünster, Austria).

CYP content of individual well-cultures was determined using the method described by Otey (2003) and Johnston et al. (2008), with minor modifications. Briefly, bacterial cells were harvested and washed twice with PBS buffer (pH 7.4) and re-suspended in 200 μl of the same buffer, containing 1 mg/ml sodium dithionite, hence obtaining a sevenfold concentrated cell-suspension. Whole-cell CYP content was determined in a flat-bottom 96-well microplate using a SpectraMax i3x microplate reader (Molecular Devices, Silicon Valley, CA, United States), measuring absorbance at 436, 450, 470, and 490 nm. Cells suspensions were then subjected to 100% CO-atmosphere for 15 min in a gas-tight bag before performing the second reading at the same wavelengths to record the reduced, CO-bound CYP absorbance. CYP contents were calculated using the equation previously mentioned (Johnston et al., 2008).

CYP1A2, 2A6, or 3A4 activities in whole-cells were assayed using the high-throughput CYP-activity assay described in our recent report (Esteves et al., 2018). Observed rate constants (*k*_*obs*_) [pmol of fluorescent product formed/(pmol of CYP per minute)] were measured. Cultures of CPR_*wt*_/CYP and CPR_*null*_/CYP were used as controls in all experiments. Control strains were obtained for each CYP (1A2, 2A6, or 3A4): one expressing CPR_*wt*_, through expression plasmid pLCM_POR_*wt*_, or with the mock plasmid pLCM (lacking *POR* cDNA), each combined with the respective human CYP expression plasmid (pCW_CYP) (Kranendonk et al., 1998; Palma et al., 2013). Clones demonstrating reaction velocities above 110%, i.e., an increase above two times background variation (5%), were selected.

### Identification of CPR-FMN-Domain Mutations

Plasmid DNA was isolated from overnight cultures of selected CPR_*mut*_/CYP clones using GeneJET Plasmid Miniprep Kit (Thermo Fisher Scientific). The cDNA encoding full length human CPR of isolated clones was extensively sequenced, using four sequencing primers (see Supplementary Table S1) for identification of FMN-domain mutations and to rule out any potential undesired additional mutations in other segments of the POR cDNA. Sequences were aligned and analyzed as described above.

### Enzyme Activity Evaluation Using Membrane Fractions

Membrane fractions of selected CPR_*mut*_/CYP clones were prepared and characterized for CYP- (CO-difference spectrophotometry), CPR- (immune-detection by western blotting) and protein-contents (Bradford assay), using previously described methods (Duarte et al., 2007; Kranendonk et al., 2008; Moutinho et al., 2012; Palma et al., 2013; Campelo et al., 2018; Esteves et al., 2018). The intrinsic electron donation capacity of the CPR-FMN-domain mutants was assessed by DCPIP reduction. These assays were performed in a buffer containing 50 mM Tris, 150 mM KCl, 10 mM NaN_3_, 0.04% Triton X-100, pH 7.5 at 37°C in the presence of 200 μM NADPH and 70 μM DCPIP. Initial rates were monitored at 600 nm using Δε_*M*_ = 21,000 M^–1^ cm^–1^ (Campelo et al., 2017).

The catalytic activities of CYP1A2, 2A6, or 3A4, sustained by CPR-FMN-domain mutants were evaluated as reported previously (Palma et al., 2010; Campelo et al., 2018). Initial velocities were measured in triplicate and plots of velocity traces *versus* substrates concentrations could be fitted according to the Michaelis-Menten equation (*r*^2^ ≥ 0.95) to determine steady-state kinetic parameters, using GraphPad Prism 5.01 Software (La Jolla, CA, United States) (Kranendonk et al., 2008; Moutinho et al., 2012; Campelo et al., 2018; Esteves et al., 2018).

### CYB5 Competition and Ionic Strength Effect

Full length human CYB5 was expressed in *E. coli* BL21-DE3 and purified as reported by Campelo et al., 2018. The effect of CYB5 and salt concentrations on CYP1A2, 2A6, or 3A4 activities, sustained by CPR-FMN-domain mutants, was assessed through microtiter plate approaches, as described previously (Campelo et al., 2018). All reaction velocities (*k*_*obs*_) were measured at least in triplicate [pmol of fluorescent product formed/(pmol of CYP per minute)].

### Multiple Alignment of CPR Mutants

Sequences of CPR proteins were downloaded from Universal Protein Resource (UniProt)^2^, aligned and analyzed (position-numbering was based on NCBI consensus sequences NM_000941.3) using the AliView software version 1.25 (Department of Systematic Biology, Uppsala University, Sweden). The alignment was performed with the MUSCLE algorithm from the AliView embedded routine.

### Structural Analysis of CPR Mutants

The structural analysis of three mutants (P117H, G144C, and A229T) was performed using the crystal structure PDB 5FA6 of the FMN-domain of human CPR_*wt*_ as the starting material. Mutations were constructed with the YASARA software^3^ and two independent molecular dynamics simulations were run at 298 K for 24 ns, using the AMBER14 force field, with 0.9% NaCl and pH 7.4. Snapshots of the simulation were taken every 100 ps. The YASARA software was used to calculate the coordinates of the average molecule for the WT and mutants and the root mean square fluctuation (RMSF) deviations calculated for each atom introduced as B factors.

### Statistical Analysis

Variance in data was analyzed through one-way ANOVA with Bonferroni’s multiple comparison test. The unpaired Student’s *t*-test was performed for calculation of the two-tailed *P*-value. The analysis was performed with 95% confidence interval using the GraphPad Prism 5.01 Software. The significance level considered in all the statistical tests was 0.05.

## Results

### Creation of CPR-FMN-Domain Mutant-Libraries

Libraries of the FMN-domain section of the cDNA of the *POR* gene carrying random mutations were generated with increasing frequencies, namely 0.9, 1.0, 1.2, 1.3, and 1.4 mutations per FMN-domain segment (Supplementary Figure S1). The mutated cDNA stretches of each set, were cloned back in the human CPR expression vector pLCM_POR (Kranendonk et al., 2008), creating five libraries for full-length CPR expression with different extents of mutagenesis restricted to the FMN-domain. Each of these random CPR-FMN-domain mutant-libraries was combined separately with three different CYP expression plasmids, for human isoforms CYP1A2, 2A6, or 3A4. CPR-FMN-domain mutants and CYPs were expressed using the dedicated *E. coli* strain, BTC, containing the specialized bi-plasmid system adequate for co-expression of CPR with representative human CYPs (Duarte et al., 2005, 2007). As such, a total of 15 BTC CPR_*mut*_/CYP libraries with CPR-FMN-domain mutant frequencies ranging from 0.9 to 1.4, were generated (Supplementary Table S2).

### Screening of CPR-FMN-Domain Mutant-Libraries

From the 15 libraries, a total of 2376 CPR_*mut*_/CYP clones were screened, using our recently developed whole-cell high-throughput activity assay (Esteves et al., 2018). With this approach, CYP-expression levels may vary from clone to clone (due to difference in expression between different wells of the microtiter plate), interfering with the detection of CPR variants, supporting increased CYP activity. Therefore rates of product formation were normalized with measured CYP expression levels from each clone. CPR_*mut*_/CYP clones were selected when presenting a 10% or more increase in turnover rates, when compared to the parental clones containing wild type CPR. This cutoff was selected based on the experimental setup in which turnover rates of the CPR_*wt*_/CYP strains demonstrated standard deviations from average values, well below 10% determined with preliminary assays (data not shown). From the 2376 CPR_*mut*_/CYP clones screened, seven were selected due to enhanced CYP activities (*k*_*obs*_ increase ranging from 1.2 to 7.25 × fold) (Supplementary Figure S2). The cDNA of CPR of each of these seven clones was subsequently sequenced to identify their FMN-domain mutations. A total of seven mutations (six single and one double mutant) were identified (Table 1).

**TABLE 1:** CYP and CPR contents of BTC membrane fractions and CPR electron donation capacity.

Membrane fractions		Protein contents			DCPIP reduction
CYP isoform	CPR form	CYP	CPR	CPR:CYP	Initial rates
		(pmol/mg protein)		ratios^a^	(min^–1^)^b^
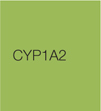	wt	54±1	4.1±1.5	1: 13	2282±239
	P117H	148±1	12.2±0.5	1: 12	2507±208
	G144C	124±2	14.1±0.2	1: 9	2233±185
	A229T	71±4	17.3±0.2	1: 4	2262±228
	CPR_null_	91±6	−	−	−

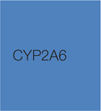	wt	139±1	10.5±1.3	1: 13	2625±285
	P117L/L125V	124±1	10.2±0.3	1: 12	2570±275
	G175D	112±6	11.3±0.5	1: 10	2508±290
	H183Y	133±1	9.4±0.1	1: 14	2544±260
	CPR_null_	178±4	−	−	−

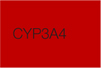	wt	83±3	19.8±0.2	1: 4	2227±201
	N151D	45±1	12.7±0.6	1: 4	2293±159
	CPR_null_	85±3	−	−	−

*(−) not determined. CYP and CPR contents are mean ± SD (technical replicates N = 3). The CPR_wt_/CYP membrane fractions were from the same batch used in our former study (Campelo et al., 2018). ^a^CPR:CYP ratios of the CPR_mut_ were not significantly different from the ones of the corresponding CPR_wt_ (P > 0.05), with each tested CYP. ^b^No significant differences were observed between measurements of DCPIP reduction in the membrane fractions studied.*

### Detailed Enzyme Activity Characterization of BTC CPR_*mut*_/CYP Clones

Membrane fractions were isolated from the selected seven CPR_*mut*_/CYP clones for confirmation of increased CYP activity and for further detailed analysis. Characterization of contents of heterologous expressed proteins from the selected seven CPR_*mut*_/CYP clones is shown in Table 1. The CPR:CYP stoichiometries measured in membrane fractions were similar to those described previously for the BTC system in our former studies, when expressing CYP1A2, 2A6 or 3A4, and comparable with the ranges found physiologically (Duarte et al., 2005, 2007; Moutinho et al., 2012; Palma et al., 2013; McCammon et al., 2016; Campelo et al., 2018; Esteves et al., 2018). Co-expression of the mutant CPRs with CYP1A2, 2A6, or 3A4 was achieved with a stoichiometry approximating the one obtained with CPR_*wt*_ for each CYP, demonstrating no significant differences (*P* > 0.05). The only exception was found for the A229T CPR variant, for which the CPR:CYP1A2 ratio was approximately three times smaller when compared to CPR_*wt*_. Thus, the data of this mutant was interpreted subsequently with caution.

**FIGURE 1:**
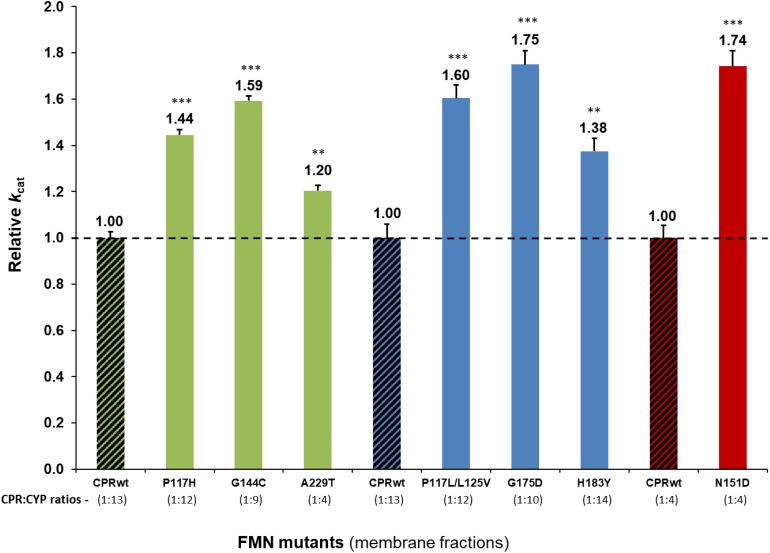
Relative turnover rates (*k*_*cat*_) (x fold) of the seven CPR-FMN mutants candidates supporting increased CYP activities, normalized by the *k*_*cat*_ demonstrated by CPR_*wt*_/CYP (determined in membrane fractions) (technical replicates *N* = 3). CPR in combination with CYP1A2 – green; 2A6 – blue; 3A4 – red; CPR_*wt*_/CYP – black stripes. ^∗∗∗^*P* < 0.0005; ^∗∗^*P* < 0.005.

Detailed analysis using membrane preparations confirmed enhanced turnover rates (*k*_*cat*_) of the seven selected CPR_*mut*_/CYP candidates, when compared to those measured with CPR_*wt*_/CYP (Figure 1 and Supplementary Table S3). To further verify if augmented reaction velocities are due to improved CPR:CYP interaction and not to altered FMN content and/or change in redox-potential of FMN in CPR_*mut*_, the reduction rates of DCPIP were measured. This assay measures the electron flow from NADPH to FAD and finally to FMN, which directly reduces DCPIP, a small chemical electron acceptor. This reaction is independent of protein:protein interaction involving the FMN-domain interface. As shown in Table 1, the DCPIP reduction rates of CPR_*mut*_ showed no significant differences when compared to CPR_*wt*_, indicating no changes in the cofactors (FAD and FMN) content and redox potential for all CPR mutants. In summary, the observed differences in CYP activities for the different CPR_*mut*_ originate only from their improved interactions with their CYP partner, with the possible exception of the A229T mutant (*vide supra*).

**FIGURE 2:**
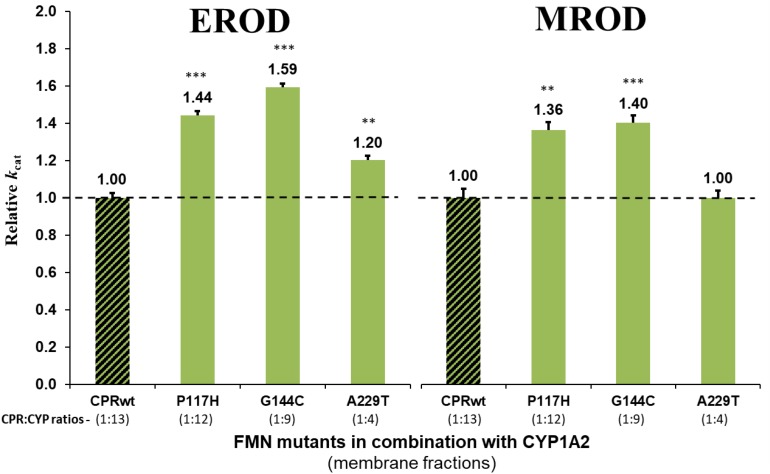
Relative turnover rates (*k*_*cat*_) (x fold) of the three CPR-FMN mutants supporting CYP1A2-mediated EROD and MROD activities. Turnover rates were determined in membrane fractions and normalized by the *k*_*cat*_ determined with CPR_*wt*_/CYP1A2 (technical replicates *N* = 3). CPR in combination with CYP1A2 – green; CPR_*wt*_/CYP – black stripes (EROD data same as presented in Figure 1). ^∗∗∗^*P* < 0.0005; ^∗∗^*P* < 0.005.

The potential effect of the substrate bound on the interaction of the CPR mutants with CYP, was subsequently tested using an additional compound with CYP1A2 and its selected CPR mutants. Comparison of relative reaction velocities of CPR_*mut*_:CYP1A2 using the substrate methoxy-resorufine with those formerly obtained with ethoxy-resorufine, are summarized in Figure 2 (see additional data in Supplementary Table S3). CPR mutants P117H and G144C stimulated significantly the CYP1A2 mediated MROD reaction albeit less pronounced as those found for EROD. The A229T CPR mutant demonstrated no significant stimulation of the MROD reaction when compared to CPR wild type, although this was observed with EROD. This might be attributed either to the CPR_*A*__229__*T*_:CYP1A2 ratio of the membrane fraction of this specific mutant, which can be a confounding factor in the assessment of the CPR:CYP interaction or, alternatively, to a different modulation in the CPR:CYP binding by a different substrate. Thus, the EROD and MROD results seems to confirm the improved CPR:CYP1A2 stimulation conferred by CPR_*P*__117__*H*_ and CPR_*G*__144__*C*_. Nevertheless, the slight variations in relative *k*_*cat*_ observed for each of the CPR_*mut*_:CYP1A2 couples, when tested with a different substrate, might be indicative that stimulation of CYP-mediated reactions by these CPR_*mut*_ may also be affected by the substrate bound.

### CYB5 Competition With CPR_*mut*_/CYP

**FIGURE 3:**
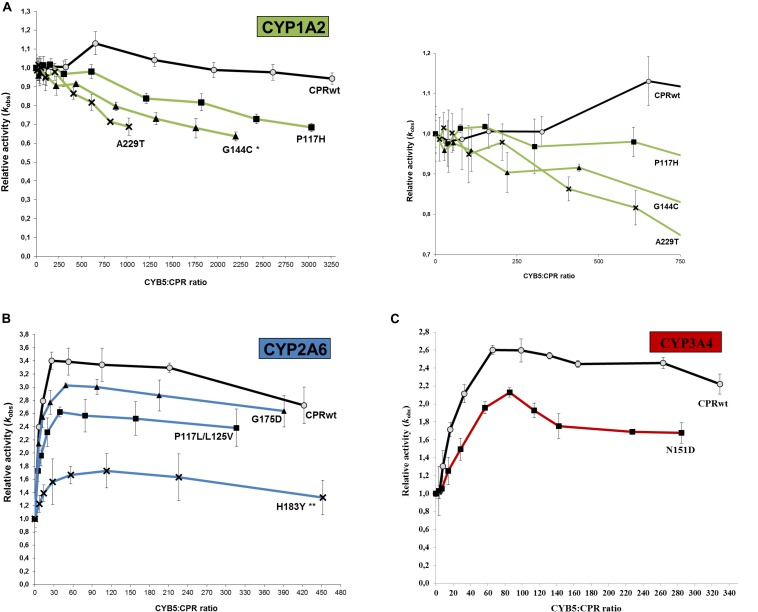
Variation of the relative CYP activities of the seven CPR-FMN mutants plotted in function of the CYB5:CPR ratio. Substrate concentrations were hold constant (5 μM ethoxyresorufin, 20 μM coumarin, or 7.5 μM dibenzylfluorescein), applying a gradient of CYB5 (0–2000, 0–800, and 0–2000 nM for CYP1A2, 2A6 and 3A4, respectively). **(A)** CYP1A2 (EROD), **(B)** CYP2A6 (C7H), and **(C)** CYP3A4 (DBODF). Observed rate constants sustained by the CPR (*k*_*obs*_) were normalized by the CYP activity without CYB5 [*k*_*obs* (__0 nM CYB__5__)_]. Normalized CYP activities represent the average of the three replicates and the error bars the standard deviation. Significant differences in the variance of CYP1A2 (*P* < 0.05) **(A)** and 2A6 (*P* < 0.005) **(B)** activities were observed, while variance of CYP3A4 activities **(C)** was close to significance (*P* = 0.06). Also, significant differences were detected in the multiple variance comparison: **P < 0.005; *P < 0.05.

CPR and CYB5 bind to CYP through largely overlapping binding sites located on the proximal side of the CYP protein, predicting that these redox partners will compete for CYP with a mutually exclusive binding (Bridges et al., 1998; Zhang et al., 2007; Waskell and Kim, 2015; Gentry et al., 2018, 2019). Improvement in binding of the different FMN-domain mutants with CYP may therefore be studied by CYB5 competition experiments, similarly to what was described in our previous report on the hinge region of CPR (Campelo et al., 2018). The relative activity ratios (k_*obs*_ mutant/k_*obs*_ wild type) were determined for the seven CPR-FMN-domain mutants with various CYB5/CPR ratios for CYP1A2, 2A6, or 3A4-mediated reactions (Figure 3). As demonstrated in our former study (Campelo et al., 2018), CYB5 has two putative effects on CYP-mediated reactions. The first one is present at low CYB5:CPR ratios and consists of a stimulatory effect caused by the second electron donation and/or allosteric effect, in a CYP and/or substrate dependent manner (Palma et al., 2013; Bart and Scott, 2017). The second one is the reverse effect: CYB5 can inhibit the CYP dependent reactions at high CYB5:CPR stoichiometries. The latter is caused by direct competition between CYB5 and CPR for the single binding site on CYP, thus blocking the first electron transfer from CPR to CYPs (Waskell and Kim, 2015) or alternatively, by direct ET from CPR to CYB5, leading to reduced transfer to CYPs and thus reduced activities. However, inhibition patterns of CYB5 on the different CYPs are isoform specific and each curve obtained with CPR_*mut*_ follow the shape of the inhibition curves acquired with CPR_*wt*_. Therefore, the inhibition pattern mediated by CYB5 cannot be attributed to the ET from CPR to CYB5. Hence, we rationalized that if a FMN-domain mutation is responsible for increased effectiveness in the docking of CPR with CYPs, it should also hamper the CYP specific CYB5 effect.

The profiles obtained from the CYB5 competition experiments showed that, in the presence of CYB5, reaction velocities of CPR_*mut*_/CYP were consistently lower than those from CPR_*wt*_/CYP (Figure 3 and Table 2). The CYP isoform differentials CYB5-effect also coincides with the knowledge that CYP3A4 and in particular CYP2A6, are much more prone for CYB5 stimulation than CYP1A2, which is less sensitive to CYB5 for activity (Duarte et al., 2005, 2007).

**TABLE 2:** Effect of CYB5 and ionic strength on CPR_*mut*_/CYP combinations.

CYP isoform	CPR form	CYB5 competition		Ionic strength	
		CYB5 effect (*%*)^a^	CYB5:CPR ratio^b^	NaCl effect (%)^a^	[NaCl] (mM)^c^
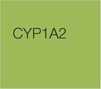	CPR_wt_	100±5	652	100±6	90
	CPR_P117H_	90±1	152	98±6	90
	CPR_G144C_	88±3	0	93±6	70
	CPR_A229T_	90±3	26	99±4	70

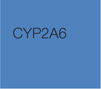	CPR_wt_	100±4	26	100±10	130
	CPR_P117L/L125V_	89±1	49	95±3	70
	CPR_G175D_	77±2	40	101±3	70
	CPR_H183Y_	51±8	113	102±2	70

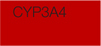	CPR_wt_	100±1	66	100±2	170
	CPR_N151D_	82±1	85	92±2	170

*^a^Maximum CYB5 or salt effect on CYP-activity. Normalized maximum enzyme activities (%) were determined by dividing the maximal turnover velocities of the CPR_mut_/CYP (k_obs (max)_ CPR_mut_) by the ones obtained of the corresponding CPR_wt_/CYP (k_obs (max)_ CPR_wt_). Values are expressed as the mean of three independent experiments ± SD. ^b^CYB5:CPR ratio at which maximum k_obs_ was reached. ^c^Salt concentration at which maximum k_obs_ was reached.*

The different CPR_*mut*_/CYP couples demonstrated lower relative CYP reaction velocities (i.e., versus the velocity without CYB5), at low and high CYB5/CPR ratios. This was particularly true for CYP2A6 and CYP3A4 (Figures 3B,C) while for CYP1A2 – as CYB5 does not have a strong stimulatory effect on this isoform – only the inhibition pattern was visible (Figure 3A and inset of Figure 3A). Overall, and not withstanding variations in the optimal stoichiometry between CYB5/CPR for activation of the CYPs activities, the stimulation (at low CYB5:CPR ratios) and inhibition (at higher CYB5:CPR ratios), are less pronounced with all CPR_*mut*_ compared to CPR_*wt*_. We thus can conclude that the binding of CYB5 to the different CYPs is probably greatly perturbed by the mutations present on the FMN domain of the various CPR_*mut*_. Collectively, the CYB5 competition data seems to be highly indicative of improved interactions of the FMN-domain mutants with the CYP isoform (in particular CYP2A6 and CYP3A4) for which they presented increased activities (Figure 1), with the potential exclusion of the A229T mutant.

### Ionic Strength Effect on CPR_*mut*_/CYP

Previously, we studied the role of ionic strength in ET from CPR to different acceptors (Campelo et al., 2017), demonstrating that the ionic strength dependency of electron flow from CPR to the acceptors is mainly determined by the salt-dependent conformational equilibrium of CPR and, to a lesser extent, by electrostatic interactions of the FMN-domain with the substrates/acceptors. More recently, we analyzed how ionic strength specifically modified the activity of CYP1A2, 2A6, and 3A4 (Campelo et al., 2018). CYP1A2 showed high, 3A4 intermediate and 2A6 relatively low sensitivity in their activities to varying ionic strength, indicative that CYP samples CPR’s open conformations in an isoform-specific manner. Additionally, our former data corroborated the study by Yun et al. (1998), that ascribed the ionic strength effect observed mostly to CPR:CYP “interaction,” while having relatively minor effects on CYP protein conformation (Yun et al., 1996).

**FIGURE 4:**
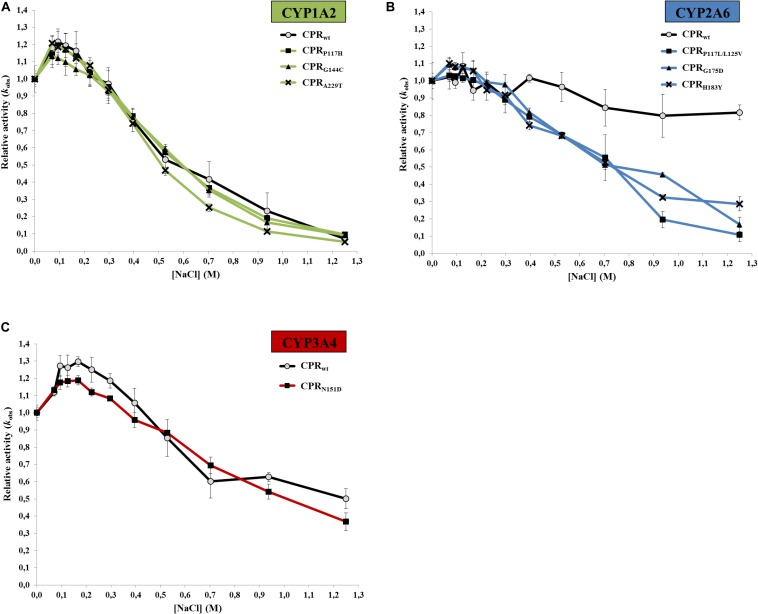
Variation of the relative CYP activities of the seven CPR-FMN mutants plotted in function of the ionic strength. **(A)** CYP1A2 (EROD), **(B)** CYP2A6 (C7H), or **(C)** CYP3A4 (DBODF). Observed rate constants sustained by the CPR (*k*_*obs*_) were normalized by the CYP activity without NaCl [*k*_*obs* (__0 nM NaCl)_]. Normalized CYP activities represent the average of the three replicates and the error bars the standard deviation (values of CPR_*wt*_/CYP were those reported in our previous study (Campelo et al., 2018).

In order to verify if the FMN-domain mutants have altered isoform specific sensitivities toward ionic strength (salt-dependent conformational equilibrium and/or electrostatic interactions with the substrates/acceptors), activities of CYPs with their specific CPR mutants were tested with various NaCl concentrations (Figure 4). The CPR mutants demonstrated very similar bell-shaped salt/activity profiles for CYP1A2 (Figure 4A) and 3A4 (Figure 4C) when compared to those obtained with wild type CPR, with a strikingly similar salt concentration at which the maximal CYP-activity is obtained (Table 2). These results may indicate that the ionic strength dependent conformational equilibria of these different CPR_*mut*_ (P117H, G144C, A229T, and N151D) are probably not altered by the mutations and thus that the effects of the mutations are probably more related to modified interactions between CYPs and CPR_*mut*_. As such, subtle changes in ionic interactions between the FMN-domain mutants and these CYPs may well be obscured by the major ionic strength effect on CPR’s open/closed dynamics.

On the contrary, this was not the case for the CPR mutants obtained with CYP2A6 (Figure 4B). With NaCl concentration ranging up to 300 mM, the three CPR_*mut*_/CYP2A6 activity profiles were analogous to CPR_*wt*_/CYP2A6 and thus the optimal concentration of NaCl (between 50 and 150 mM) to obtain maximal velocity of CYP2A6 is undistinguishable between CPR_*wt*_ and CPR_*mut*_. However, at ionic strength above 300 mM, all CPR_*mut*_ displayed increased sensitivities to ionic strength which translated into lower CYP2A6 activities. The nature of the CPR mutations, for which there is no evident pattern of electrostatic change (P to L, L to V, G to D, or H to Y), together with the fact that the increased sensitivities to ionic strength are specific to CPR_*mut*_ that have augmented activities with CYP2A6, may again indicate specific effects of the mutations on the recognition with CYP2A6 although effects on the open/closed dynamics of CPR cannot be totally excluded based on our former observations.

## Discussion

**FIGURE 5:**
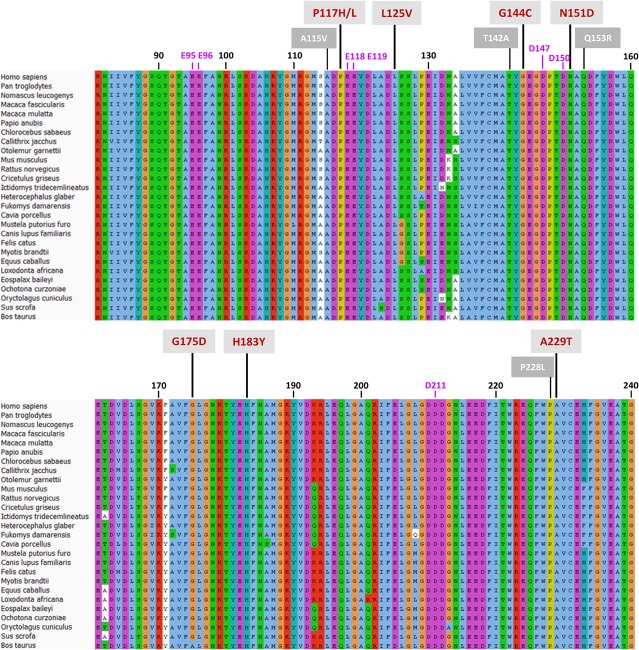
Protein sequence alignment of the CPR-FMN-domain of 27 mammals. Sequences of CPR proteins were downloaded and aligned with Universal Protein Resource (UniProt) (www.uniprot.org), using the Clustal X color scheme for amino acid alignments. Residue numbering (in black) is according to human CPR NCBI consensus protein sequence NP_000932.3. Mutations are indicated with red numbering (mutations selected in this study) or white numbering (natural variants of FMN-domain with CYP isoform specific effects).

Little is known about the mechanism by which CPR selects one of its many ET partners (Waskell and Kim, 2015). It seems clear that, together with the open/close dynamics, the FMN-domain plays a central role in the docking of CPR with CYP in the ER membrane. This raises the question how CPR, primarily through its FMN-domain, enables the affinity sampling by such a wide range of structurally diverse CYP-isoforms. This was the issue addressed by this study, using CPR variants of the FMN-domain, obtained by means of random mutagenesis. Although the size of the mutant libraries was nowhere near that of the sequence space, the success in identifying FMN-domain mutated proteins supporting a gain in CYP-activity, is indicative of a target sequence carrying densely packed functional features (Skandalis et al., 1997). This is highlighted by the high degree of conservation of this domain among species, particularly mammals (Figure 5 and Supplementary Table S4).

### FMN-Domain Mutations and Their CYP-Dependent Effect

All selected FMN-domain mutations induced an increase in reaction velocity (Figure 1). The obtained CYB5 competition data seems to be indicative of an improved interaction of the FMN-domain mutants with their respective CYPs, in particular those for CYP2A6 and 3A4 and less clearly for CYP1A2, in particular for CPR mutant A229T. The CYB5 effect is rather small for CPR_*wt*_ and CPR_*mut*_ combined with CYP1A2, which may hinder the detection of possible binding differences between wild type and mutant CPRs. Indeed the ability of CYB5 to bind CYP and to compete with CPR has recently been demonstrated by NMR studies to be dependent on multiple factors including the effect of the substrate bound and membrane anchoring (Gentry et al., 2018, 2019). In this respect, our data regarding the use of two different CYP1A2 substrates seem to indicate that the substrate bound may have a modulating role in CPRmut binding. This could be due to the fact that CYP’s substrate binding site is considered to be rather malleable, and due to its close proximity may influence the architecture of the CPR binding site.

To assess if improved interactions of the FMN-domain mutants with their respective CYPs could be primarily caused by increased ionic interactions, changes in the salt profiles were also examined. Our data showed that this was mainly the case for the CPR mutants selected with CYP2A6. CYB5’s binding site on CYP is similar and overlaps with that of CPR. In addition, it is mainly guided by ionic interactions through CYB5 acidic surface residues (Waskell and Kim, 2015). Interestingly, CPR interactions with the most CYB5-sensitive of the three CYPs (CYP2A6, Figure 3B), demonstrated a strong difference in the ionic strength activity dependence, being poorly or very sensitive to increased NaCl concentrations with CPR_*wt*_ or CPR_*mut*_, respectively (Figure 4B). Conversely, CYP1A2 and 3A4 demonstrated high and intermediate ionic strength sensitivities that are indistinguishable between CPR_*wt*_ and CPR_*mut*_ and these two CYPs are respectively poorly or intermediately stimulated by CYB5. Therefore, it seems that CYB5’s capability to compete with CPR for CYP-binding (i.e., ability to stimulate) depends partly on the level of ionic interactions in the CPR:CYP complex, which our former (Campelo et al., 2018) and current data demonstrated to be CYP-isoform dependent. Dependence of CPR activities on ionic strength is in part due to changes in the CPR conformational equilibrium. The superimposable salt profiles for the various CPR mutants specific for CYP1A2 and CYP3A4 might indicate that the effects of mutations: (i) do not have impact in the CPR conformational equilibrium and/or, (ii) might be attributed either to a change in salt interactions that could be obscured by the large ionic strength dependency of the CYPs activities or, alternatively, to improved binding through non-ionic interactions. Improved ionic strength interactions of FMN-domain mutants obtained with CYP2A6 (P117L/L125V, G175D, and H183Y) seem to be clear for the G175D mutant, creating an additional negative charge. However this is not directly obvious for the remaining mutations (P117L/L125V and H183Y). Still, subtle structural alterations induced by mutations may modulate charged residues in their vicinity to come into play. Small deviations might be translated in substantial structural effects (Waskell and Kim, 2015).

### Mutations Co-localized With Structural Features Involved in CPR:CYP Interaction

**FIGURE 6:**
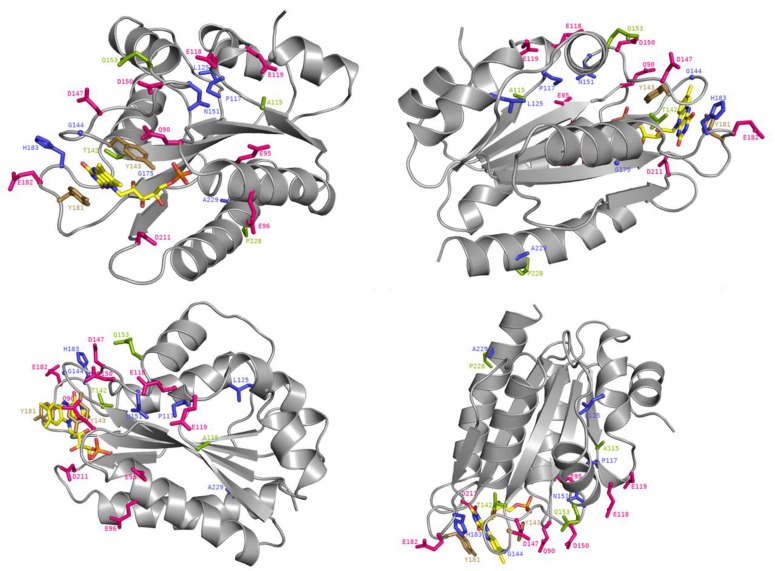
Crystal structure of the FMN-domain of human CPR (PBD 3QFR) from four different angles. Specific residues are depicted as stick with the following color code: magenta for acidic residues formerly indicated to be involved in CPR:CYP interactions, blue for generated mutations, green for mutated residues in natural occurring CPR variants with CYP isoform specific effects and gold for FMN-binding tyrosines. The FMN molecule is colored in yellow with the exceptions of its nitrogen atoms (in blue), its oxygen atoms (in red) and its phosphorus atom (in orange).

Some of the identified mutations seem to cluster with patches of charged residues of the FMN-domain (Figure 5). For example, positions P117, N151, and G144 are in close vicinity to negatively charged residues D116, E118, E119, E145, D147, and D150 (Figure 6) which have been formerly suggested to play a role in CPR:CYP interactions (Shen and Strobel, 1994; Wang et al., 1997; Zhao et al., 1999; Hamdane et al., 2009; Hong et al., 2010; Jang et al., 2010). Additionally, two mutated positions (G144 and H183) were found adjacent to the conserved tyrosine residues, Y143 and Y181, considered to be directly involved in FMN binding (Shen et al., 1989; Marohnic et al., 2010; Xia et al., 2011) (Figure 6). The G144C and H183Y substitutions may induce subtle alterations in the surface presentation of this cofactor, improving ET for a specific CYP by, e.g., shortening the distance to the heme, although alteration of relative midpoint potential of the FMN moiety by these mutations cannot be excluded. Still our DCPIP reduction data (Table 1) seem to indicate that this is not the case. Alternatively, these substitutions may lead to a reorientation of the position of FMN, which may directly benefit physico-chemical interaction of the cofactor itself with its redox partner, as it has been suggested for FMN in a plant ferredoxin when interacting with its redox-partner (Frago et al., 2010).

The functionality of the mutated positions seems to be confirmed by co-localization of mutations found in several natural occurring CPR variants (A115V, T142A, Q153R, and P228L) (Figure 5), which have been suggested to modulate the CPR:CYP interaction and have CYP isoform specific effects (reviewed in, Pandey and Flück, 2013; Burkhard et al., 2017). Interestingly, several of our identified positions are highly conserved among species. Residues T142, G144, N151, and G175 demonstrated > 90% conservation in an alignment of 1221 CPR sequences of different organisms, with 100% conservation of G144 (Supplementary Table S4), the latter suggested to form an interaction with the FMN moiety (Rwere et al., 2016). The only FMN-domain mutant that does not seemingly fall in the categories mentioned above is A229T. Some doubts remain regarding this mutant as it was expressed with a CPR/CYP stoichiometry in favor of CPR, potentially confounding the selection for improved CYP catalysis. However, this residue is directly adjacent to the P228L substitution found in a natural occurring CPR variant, which has been demonstrated to have a CYP-isoform differential effect, as indicated above (some additional information on the positions of the selected FMN-domain mutants is given in Supplementary Material).

### Mutations Promote Subtle Deviations Within the FMN-Domain

**FIGURE 7:**
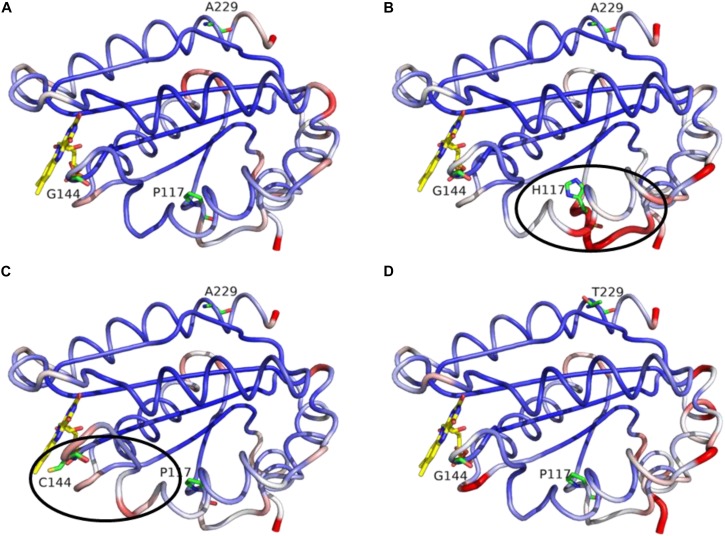
MD simulation outcome: RMS deviations for α-carbons of the FMN-domain of CPR_*wt*_ **(A)**, CPR_*P*__117__*H*_ **(B)**, CPR_*G*__144__*C*_ **(C)**, and CPR_*A*__229__*T*_ **(D)**. The figure was prepared with the PyMol software 2.3 (pymol. org/2), using the putty function to trace the ribbon. A gradient color from blue to white to red depicts the RMSF values of the α-carbons (low to medium to high RMSF values). Stable α-carbons – blue; Changed α-carbons – red; FMN and side chains of CPR_*mut*_ represented in colored sticks. α-carbon backbones with subtle adjustments of the side chains caused by CPR_*mut*_ are evidenced in black ellipses.

In regard to the CPR mutations selected with CYP1A2, we decided to assess the potential role of the three positions through molecular dynamics simulations. The latter presented no major changes in the overall shape or geometry of the FMN-domain, as indicated by the equivalent RMS deviations for α-carbons between CPR_*wt*_ and CPR_*mut*_ (Figure 7), except for two subtle changes in loop positioning’s by the G144C substitution (compare Figures 6A and 6C) and the P117H substitution (compare Figures 6A and 6B). The relative rigidity of the Rossman fold and involvement of multiple residues on several loops of the FMN domain upon interaction with CYP has recently been reported by elegant NMR studies by the group of Ayyalusamy Ramamoorthy (Prade et al., 2018; Gentry et al., 2019). Our data implies that the changes induced by the mutants probably propagate to the sidechains with minor changes on the backbone. Such effect is not unusual for the CYP enzyme complex, as recently described for the CYP1A1 enzyme, in which substrate binding was accommodated by minimal perturbation of the α-carbon backbone with subtle adjustments of the side chains (Bart and Scott, 2018). Additionally, a single amino acid change in a redox partner of CYP106A2 induced a considerable increase in substrate conversion by this bacterial CYP, with a minimal conformational perturbation (Sagadin et al., 2019).

## Conclusion

Our results seem to suggest that every CYP is interacting with CPR in a specific manner, making use in an isoform specific mode, of docking elements, i.e., a composite of binding motifs of the FMN-domain. Therefore, the capacity of CPR to interact with so many CYPs can be attributed to the presence of different open conformations which are affinity-sampled through the different binding motifs, by which each binding partner can be supplied with electrons. Altering or modifying the composition of open conformations (as we have shown recently in Campelo et al., 2018) and/or modifying these binding motifs, i.e., in the vicinity or within, will alter interaction with a particular redox partner. It is likely that some of these binding motifs are more common, i.e., shared among different CYPs, while others are more relevant for a specific CYP. Some of these FMN binding motifs might not yet been identified, and we hypothesize that one may be present around the P228-L229 positions.

Natural occurring variations of these CPR motifs may alter CYP-activity profiles, with the potential of causing specific genetic susceptibilities and pathologies. Drugs are metabolized for their excretion, frequently with involvement of multiple CYP isoforms. Natural occurring FMN-domain variants of these motifs may change the relative involvement of CYP isoforms, leading to altered metabolic profiles. In turn, this may potentially be related to lack of efficacy in drug treatment and/or adverse drug reactions. Likewise, genetic variations of residues forming the FMN-domain motifs may influence specific CYPs involved in steroidogenesis. Naturally occurring variants of the *POR* gene encoding CPR have been linked to a broad spectrum of human diseases, ranging from severe skeletal malformation and perturbed steroidogenesis (Antley-Bixler syndrome; OMIM reference # 201750) to phenotypical normal individuals with, e.g., infertility (Tee et al., 2011). Former studies have made suggestions how mutations found in several natural occurring variants might interfere with the CPR:CYP interactions (reviewed in, Pandey and Flück, 2013; Burkhard et al., 2017). Our data seem to further underpin mechanistically these suggestions and the pathological consequences of these *POR* mutations.

## Author’s Note

The authors dedicate this research to the memory of Dr. Henry W. Strobel.

## Data Availability Statement

All datasets generated for this study are included in the article/Supplementary Material.

## Author Contributions

FE, GT, and MK: planning experiments. FE, DC, BG, and SB: performed experiments. All authors: data analysis and interpretation, writing, reviewing, editing of the manuscript, and approval to the final version of the manuscript. GT and MK: coordination and funding acquisition. All authors reviewed and gave approval to the final version of the manuscript.

## Conflict of Interest

The authors declare that the research was conducted in the absence of any commercial or financial relationships that could be construed as a potential conflict of interest.

## Footnotes

1 https://drnelson.uthsc.edu/fuguhum.tree.pdf

2 www.uniprot.org

3 www.yasara.org

## Abbreviations

C7H: coumarin 7-hydroxylation
CPR: NADPH cytochrome P450 oxidoreductase
CYB5: cytochrome b5
CYP: cytochrome P450
DBODF: dibenzylfluorescein O-debenzylation
ET: electron transfer
ER: endoplasmic reticulum
EROD: ethoxy-resorufin O-deethylation
FAD: flavin adenine dinucleotide
FMN: flavin mononucleotide
MD: molecular dynamics
MROD: methoxy-resorufine O-demethylation
Mut: mutant
wt: wild type
