# Corrigendum: The Role of the FMN-Domain of Human Cytochrome P450 Oxidoreductase in Its Promiscuous Interactions With Structurally Diverse Redox Partners

**DOI:** 10.3389/fphar.2020.00773

**Published:** 2020-05-21

**Authors:** Francisco Esteves, Diana Campelo, Bruno Costa Gomes, Philippe Urban, Sophie Bozonnet, Thomas Lautier, José Rueff, Gilles Truan, Michel Kranendonk

**Affiliations:** ^1^Centre for Toxicogenomics and Human Health (ToxOmics), Genetics, Oncology and Human Toxicology, NOVA Medical School, Faculty of Medical Sciences, Universidade NOVA de Lisboa, Lisbon, Portugal; ^2^Centre National de la Recherche Scientifique, Institut National de la Recherche Agronomique, Institut National des Sciences Appliquées de Toulouse, Toulouse Biotechnology Institute, Université de Toulouse, Toulouse, France

**Keywords:** NADPH cytochrome P450 oxidoreductase (CPR), cytochrome P450 monooxygenases (CYP), FMN-domain, binding motifs, redox partner, protein–protein interaction, electron transfer

In the original article, the reference to the study by Gideon et al. (2012) was erroneously used in the second paragraph of the *Introduction*. It has now been removed.

The authors apologize for this error and state that this does not change the scientific conclusions of the article in any way. The original article has been updated.
