# Supplementary material for: The Role of the FMN-Domain of Human Cytochrome P450 Oxidoreductase in Its Promiscuous Interactions With Structurally Diverse Redox Partners
Source: Front Pharmacol. 2020 Mar 18;11:299. doi: 10.3389/fphar.2020.00299 (PMC7094780; doi:10.3389/fphar.2020.00299)
Supplement: Supplementary file 1 [file Data_Sheet_1.pdf]

frontiers

Supplementary Material

The Role of the FMN-domain of Human Cytochrome P450 Oxidoreductase in its Promiscuous Interactions with Structurally Diverse Redox Partners

Francisco Esteves[1*], Diana Campelo[1], Bruno Costa Gomes[1], Philippe Urban[2], Sophie Bozonnet[2], Thomas Lautier[2], José Rueff[1], Gilles Truan[2], and Michel Kranendonk[1*]

Author affiliations: [1]Center for Toxicogenomics and Human Health (ToxOmics), Genetics, Oncology and Huma Toxicology, NOVA Medical School/Faculty of Medical Sciences, Universidade NOVA de Lisboa, Lisbon, Portugal; [2]LISBP, Université de Toulouse, CNRS, INRA, INSA, Toulouse, France.

[*] **Corresponding authors:**

Francisco Esteves

Address: Center for Toxicogenomics and Human Health (ToxOmics), Genetics, Oncology and Human Toxicology, NOVA Medical School/Faculty of Medical Sciences, Universidade NOVA de Lisboa, Edifício CEDOC II | Room 2.2 | Rua Câmara Pestana, 6 |1150-082 LISBOA, Portugal.

E-mail: francisco.esteves@nms.unl.pt

Phone: (+351) 218 803 101

Michel Kranendonk

Address: Center for Toxicogenomics and Human Health (ToxOmics), Genetics, Oncology and Human Toxicology, NOVA Medical School/Faculty of Medical Sciences, Universidade NOVA de Lisboa, Edifício CEDOC II | Room 2.2 | Rua Câmara Pestana, 6 |1150-082 LISBOA, Portugal.

E-mail: michel.kranendonk@nms.unl.pt

Phone: (+351) 218 803 101

SUPPLEMENTARY TABLES

Supplementary Table S1 | Sequences of oligonucleotides used for error-prone PCR (epPCR) and direct sequencing.

Assays	Primer	Sequence (5’-3’)
**epPCR and direct sequencing of *FMN*-domain**	AdRandom POR Fw	CAGACATTGACCTCCTCTGTCAGAG
	AdRandom POR Rv	GACGAGAGCAGAGTCGTTGGCTGG
**total *POR* cDNA direct sequencing**	POR_Fw1	AACCAGCTGGGCAAAATCCT
	POR_Rv1	CTGCCGCCAGGCAAATTCTG
	POR_Fw2	AATTCTAATGGGAGACTCCC
	POR_Rv2	CGGCGGGTTGGTGATGTCCA

Supplemental Table S2 | Screening of mutant libraries of the CPR's FMN-domain produced by random mutagenesis and BTC CPR_{mut}/CYP clones candidates.

**CYP isoform (CPR_{mut}/CYP clones)**	Mutation frequency (Mutation/FMN-domain fragment [a])					
	**0.9**	**1.0**	**1.2**	**1.3**	**1.4**	**Total (%)**
**CYP1A2**	0/176 [b]	0/176	**1**/176	**1**/264	**1**/176	**3**/968 (0.31 %)
**CYP2A6**	0/176	0/176	0/176	**2**/176	**1**/176	**3**/880 (0.34 %)
**CYP3A4**	0/88	**1**/176	0/88	0/88	0/88	**1**/528 (0.19 %)

[a] used FMN-domain: positions 81-248 according to CPR consensus sequences NP_000932.3; [b] Number of positive clones with increased activity (k_{obs}) relative to the ones observed with CPR_{wt}.

Supplemental Table S3 | Kinetic steady-state parameters of CYP1A2, 2A6 and 3A4-activities.

CYP isoform	CPR form	k_{cat} (product formed pmol.min^{-1}.pmol CYP^{-1})	K_M (µM)
CYP1A2 (EROD)	**wt**	0.54 ± 0.01	1.75 ± 0.11
	**P117H**	0.78 ± 0.01 ***	0.64 ± 0.03 ***
	**G144C**	0.86 ± 0.01 ***	0.82 ± 0.04 ***
	**A229T**	0.65 ± 0.01 ***	0.92 ± 0.04 ***
CYP1A2 (MROD)	**wt**	1.03 ± 0.04	0.68 ± 0.05
	**P117H**	1.40 ± 0.03 ***	0.38 ± 0.02 **
	**G144C**	1.44 ± 0.03 ***	0.44 ± 0.02 ***
	**A229T**	1.03 ± 0.02	0.40 ± 0.02
CYP2A6 (C7H)	**wt**	0.96 ± 0.04	1.57 ± 0.24
	**P117L/L125V**	1.54 ± 0.06 ***	2.18 ± 0.27 *
	**G175D**	1.68 ± 0.07 ***	2.37 ± 0.31 *
	**H183Y**	1.32 ± 0.05 **	1.18 ± 0.25
CYP3A4 (DBODF)	**wt**	2.60 ± 0.10	0.89 ± 0.13
	**N151D**	4.53 ± 0.24 ***	1.16 ± 0.19

Control experiments with CPR_{null}/CYP membrane fractions did not show any detectable activities with the respective substrates. Kinetic parameters values of the CPR_{mut}/CYP were compared with the ones of the CPR_{wt}/CYP applying the unpaired *t* test (technical replicates N=3).

*** $P < 0.0005$

** $P < 0.005$

* $P < 0.05$

Supplemental Table S4 | Analysis of the multiple sequence alignment of the FMN-domain of CPR proteins using a database of 1221 sequences from different organisms.

CPR	Residues in FMN-domain										
	**115**	**117**	**125**	**142**	**144**	**151**	**153**	**175**	**183**	**228**	**229**
**Human** [a]	**A**	**P**	**L**	**T**	**G**	**N**	**Q**	**G**	**H**	**P**	**A**
**Database** (amino acid frequencies in 1221 CPR sequences of different species) [b]	**A** (66)	**L** (49)	**L** (68)	**T** (99)	**G** (100)	**N** (93)	**V** (38)	**G** (93)	**H** (79)	**P** (38)	**A** (45)
	**V** (14)	**P** (37)	**E** (16)	**S** (1)	-	**G** (3)	**A** (23)	**A** (6)	**F** (11)	**E** (23)	**E** (20)
	**C** (8)	**I** (7)	**I** (9)	-	-	**S** (2)	**M** (18)	**S** (1)	**Y** (7)	**A** (14)	**S** (13)
	**I** (6)	**V** (4)	**F** (2)	-	-	**P** (1)	**Q** (11)	-	**Q** (1)	**T** (7)	**T** (5)
	**L** (3)	**H** (2)	**A** (1)	-	-	**D** (1)	**N** (2)	-	**K** (1)	**K** (6)	**V** (5)
	**G** (1)	**F** (2)	**M** (1)	-	-	-	**I** (1)	-	-	**D** (5)	**K** (3)
	**T** (1)	-	**P** (1)	-	-	-	**S** (1)	-	-	**S** (2)	**L** (2)
	-	-	**V** (1)	-	-	-	**L** (1)	-	-	**V** (1)	**H** (2)
	-	-	-	-	-	-	**D** (1)	-	-	**N** (1)	**I** (2)
	-	-	-	-	-	-	**E** (1)	-	-	**Q** (1)	**D** (1)
	-	-	-	-	-	-	**T** (1)	-	-	-	**M** (1)

Sequence differences studied in detail are the ones from the generated mutations (P117, L125, G144, N151, G175, H183, A229) and naturally occurring variants (A115, T142, Q153, P228) found to be aligned in two axes, as described in Figure 4. Residues are classified and colored according to chemical character: **black**, hydrophobic amino acids; **red**, acidic amino acids; **blue**, basic amino acids; and

green, polar amino acids. The amino acid alterations coincident with the ones detected in the present study are underlined.

[a] Human CPR NCBI consensus protein sequence NP_000932.3.

[b] Database from UniProt Knowledgebase (UniProtKB); only amino acids with frequencies ≥ 1% were described.

SUPPLEMENTARY FIGURES

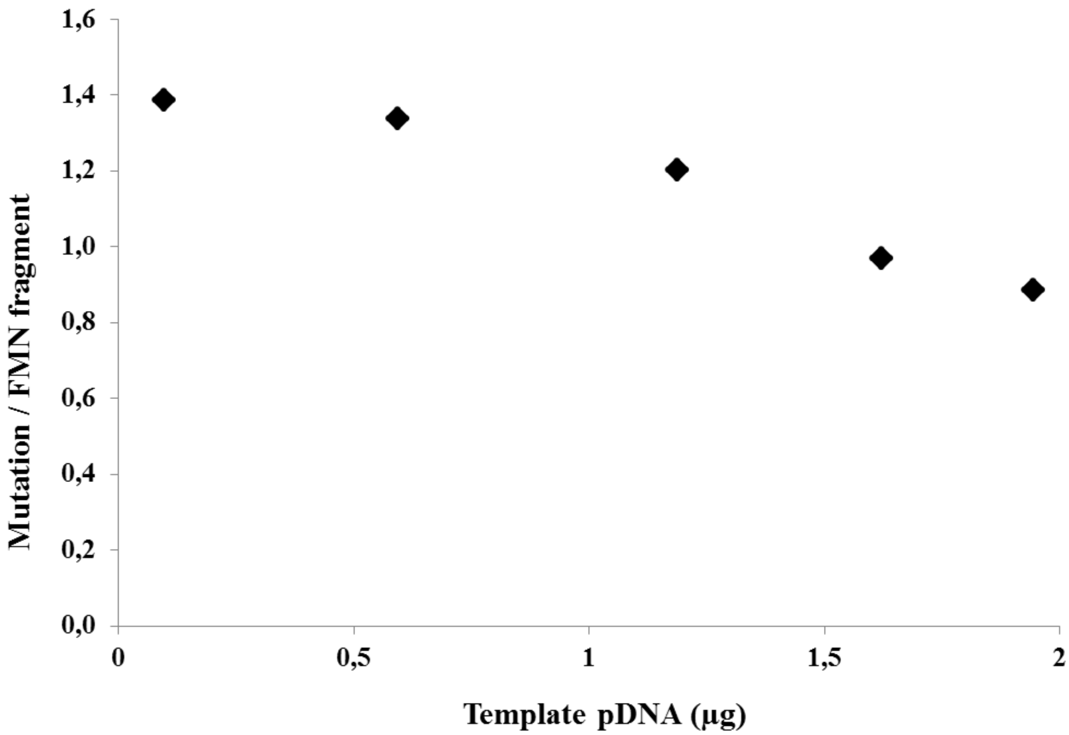

Supplemental Figure S1 | Mutation frequencies of the five CPR-FMN mutant pools constructed using epPCR.

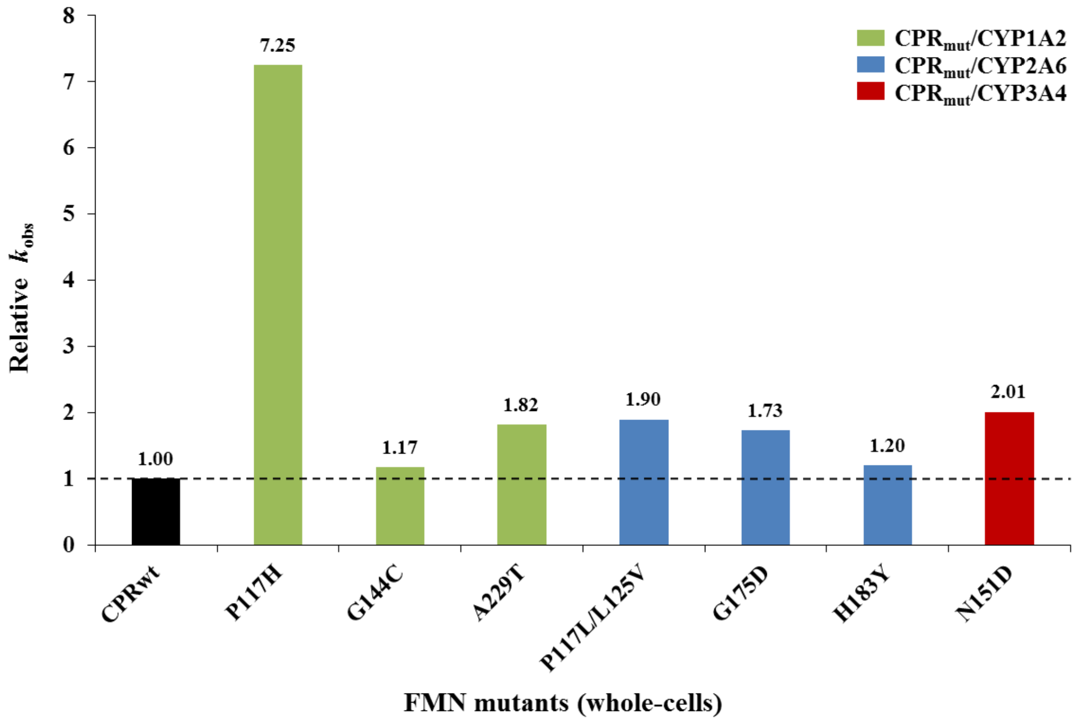

Supplemental Figure S2 | Normalized enzymatic activities (Relative observed rate constants, k_{obs}) (x fold) of the seven BTC CPR$_{mut}$/CYP clones demonstrating increased CYP activities in the whole-cell HT activity assay. CPR in combination with CYP1A2 (EROD); 2A6 (C7H); 3A4 (DBODF). Maximum turnover velocities sustained by the CPR$_{mut}$ (k_{obs} CPR$_{mut}$) were normalized by the CYP activity sustained by the CPR$_{wt}$ (k_{obs} CPR$_{wt}$). The control strains BTC CYP1A2, 2A6, or 3A4 did not show any detectable activities with the respective substrates, validating the data obtained through the screening assays.

Additional information on selected FMN-domain mutants

The CPR_{N151D} mutation that increased CYP3A4 activity is located near the surface, close to two acidic residues (D150 and D154) and hydrophilic residues (T149, Q153) (**Figure 5**). The corresponding asparagine residue in rat CPR was described to be in direct contact with CYP2B4 proximal site in a CPR-FMN-domain:CYP2B4 model (Nicolo et al., 2010; Waskell and Kim, 2015). The N151D substitution, by adding another charge onto the surface, will strongly impact the electrostatic potential of this particular position. While this modification is not really translated in the salt profile of CYP3A4 activity with CPR_{N151D}, the CYB5 profile clearly demonstrates an increase in CPR binding which is probably mediated by the electrostatic potential change.

For the mutations involved in CYP2A6, the situation is slightly more complex, as two positions are on the surface and two are embedded in the interior of the FMN-domain (**Figure 5**). Considering the strong CYB5 and salt effects of the mutants, it is probable that these positions affect in some manner the electrostatic potential (i.e. the charged surface residues positions) of the FMN-domain, resulting in an increase of the affinity between CPR and CYP2A6. Three out these four positions (P117, L125 and H183) are close to negatively charged residues present at the surface, reinforcing the hypothesis of probable electrostatic changes induced by the mutations. Interestingly, substitution of the Proline-117 selected with CYP2A6 (P117L) was also selected for an increased activity when combined with CYP1A2 when substituted with histidine (P117H). The corresponding proline in rat CPR was identified previously to be directly in contact with CYP2B4 (Nicolo et al., 2010; Pandey and Flück, 2013). These various results point to a putative role of this residue in the CPR open/close equilibrium: P117 could interact with the closely conserved negative patches (D116 and E118) and thus, mutations at this positions might modify subtle positioning of the two acidic residues, resulting in a direct interference in the interaction of the FMN-domain with specific CYP isoforms, through changes in the conformational equilibrium of CPR or to the FMN to CYPs interactions. Still, the results from P117L/L125V have to be analyzed carefully due to the difficulty in determining the effect of each of the mutations, *per se*.
